# Changes in Arterial Stiffness in Response to Blood Flow Restriction Resistance Training: A Narrative Review

**DOI:** 10.3390/jcm12247602

**Published:** 2023-12-10

**Authors:** Ioana Mădălina Zota, Cristina Mihaela Ghiciuc, Doina Clementina Cojocaru, Corina Lucia Dima-Cozma, Maria Magdalena Leon, Radu Sebastian Gavril, Mihai Roca, Alexandru Dan Costache, Alexandra Maștaleru, Larisa Anghel, Cristian Stătescu, Radu Andy Sascău, Florin Mitu

**Affiliations:** 1 Department of Medical Specialties I, Faculty of Medicine, Grigore T. Popa University of Medicine and Pharmacy, 700111 Iași, Romania; ioana-madalina.chiorescu@umfiasi.ro (I.M.Z.); cozma.dima@umfiasi.ro (C.L.D.-C.); maria.leon@umfiasi.ro (M.M.L.); sebastian.gavril@umfiasi.ro (R.S.G.); mihai.c.roca@umfiasi.ro (M.R.); dan-alexandru.costache@umfiasi.ro (A.D.C.); alexandra.mastaleru@umfiasi.ro (A.M.); larisa.anghel@umfiasi.ro (L.A.); cristian.statescu@umfiasi.ro (C.S.); radu.sascau@umfiasi.ro (R.A.S.); florin.mitu@umfiasi.ro (F.M.); 2 Pharmacology, Clinical Pharmacology and Algeziology, Department of Morpho-Functional Sciences II, Faculty of Medicine, Grigore T. Popa University of Medicine and Pharmacy, 700111 Iași, Romania; 3 Academy of Medical Sciences of Romania, Ion C. Brătianu Boulevard No 1, 030167 Bucharest, Romania

**Keywords:** blood flow restriction training, vascular stiffness, pulse wave velocity, vascular aging, resistance training

## Abstract

Arterial stiffness naturally increases with age and is a known predictor of cardiovascular morbimortality. Blood flow restriction (BFR) training involves decreasing muscle blood flow by applying a strap or a pneumatic cuff during exercise. BFR induces muscle hypertrophy even at low intensities, making it an appealing option for older, untrained individuals. However, BFR use in patients with cardiovascular comorbidities is limited by the increased pressor and chronotropic response observed in hypertensive elderly patients. Furthermore, the impact of BFR on vascular function remains unclear. We conducted a comprehensive literature review according to PRISMA guidelines, summarizing available data on the acute and long-term consequences of BFR training on vascular function. Although evidence is still scarce, it seems that BFR has a mild or neutral long-term impact on arterial stiffness. However, current research shows that BFR can cause an abrupt, albeit transient, increase in PWV and central blood pressure. BFR and, preferably, lower-body BFR, should be prescribed with caution in older populations, especially in hypertensive patients who have an exacerbated muscle metaboreflex pressor response. Longer follow-up studies are required to assess the chronic effect of BFR training on arterial stiffness, especially in elderly patients who are usually unable to tolerate high-intensity resistance exercises.

## 1. Introduction

Resistance training in addition to aerobic exercise is associated with lower cardio-vascular risk and all-cause mortality [1]. Current cardiovascular prevention guidelines recommend a resistance training protocol consisting of 1–3 sets of 8–12 repetitions at 60–80% of the patient’s repetition maximum (1RM), with 8–10 various exercises involving different major muscle groups performed at least 2 days per week [1,2]. However, elderly patients with associated osteoarthritis and cardiovascular disease are unable to withstand high mechanical stress. Such patients are usually prescribed lower-resistance training regiments, 40–50% of 1RM, along with a greater number of repetitions per set (10–15) [3]. However, exercise intensities below 70% of 1RM usually fail to produce significant muscle hypertrophy or strength gain, and several studies have approached the use of low-intensity BFR (blood flow restriction) training as an alternative strength training modality in patients with stable coronary artery disease [4,5].

Resistance training with BFR involves decreasing muscle blood flow by applying a strap or a blood pressure cuff during exercise. BFR allows effective training of skeletal muscles at lower intensities, making it suitable for untrained subjects and patients with orthopedic comorbidities [6], with muscle hypertrophy occurring even at low-intensity training (20% of 1RM) [7]. However, this technique is rarely used in patients with cardiovascular comorbidities due to safety concerns [2].

Arterial stiffness is a pivotal element in the pathogenesis of cardiovascular disease, considered an independent predictor of cardiovascular mortality and event risk [8]. Arterial stiffening is naturally associated with aging, but is accelerated in the presence of respiratory, metabolic, and cardiovascular comorbidities. Vascular stiffness is usually assessed as the aortic pulse wave travel velocity (PWV), but AIx is also accepted as a surrogate arterial stiffness parameter [9].

In healthy populations, regular exercise has a beneficial impact on vascular function. However, the acute and chronic effects of training seem to vary with different types of exercise modalities, especially in patients with coexisting health issues. Aerobic training reduces central arterial stiffness, but more recently, HIIT (high-intensity interval training) seems to have a more pronounced beneficial impact on endothelial function and arterial stiffness, mediated by the upregulation of the arterial endothelial nitric oxide synthase [10]. On the other hand, previous studies have shown conflicting impacts of resistance training on arterial stiffness [11,12]. Current evidence suggests that while high-intensity resistance training increases PWV [11], lower training intensities have a beneficial impact on vascular stiffness [12]. However, low-intensity resistance training does not correct sarcopenia, an issue which can easily be addressed with the use of BFR. Several systematic reviews and meta-analyses have analyzed the impact of low-intensity BFR training on vascular stiffness. However, they did not include all available studies (due to insufficient reported data and significant variations in protocol [13]) and provided divergent results [14,15,16,17]. For instance, Maga et al. [13] did not report significant differences in changes between BFR and non-BFR training, but included both aerobic and resistance BFR training protocols in their meta-analysis. While Pereira et al. [17] found no significant difference between low-intensity BFRE and high-intensity non-BFRE, regarding PWV, the meta-analysis conducted by Liu et al. [14] reported that BFR resistance training is more effective for regulating arterial compliance compared to traditional RT. Contrary to the results of Amorim et al. [16], another recent meta-analysis [15] showed that low-resistance BFR training in older adults will improve not only CAVI and ABI, but also flow-mediated dilation.

As previous studies have been inconsistent, the scope of this review is to summarize all current evidence regarding the impact of BFR resistance training on arterial stiffness parameters.

## 2. Materials and Methods

The population targeted in the current review are patients of all ages, with and without cardiovascular comorbidities, undergoing arterial stiffness assessment. The primary intervention was BFR resistance training, either isolated or compared to high-load resistance training or controls (no training).

### 2.1. Electronic Search Strategy

We conducted a comprehensive literature review of the articles currently available in the EMBASE, MEDLINE and PubMed databases, according to PRISMA guidelines. We used the following keywords: “blood flow restriction”, “blood flow occlusion”, “KAATSU training”, “arterial stiffness”, “PWV” and “pulse wave velocity”. This review was carried out according to the Preferred Reporting Items for Systematic Review and Meta-Analysis (PRISMA) checklist [18]. We applied the following selection criteria:

- Study type: retrospective, cross-sectional or prospective analysis, case reports and case series;
- Language: English;
- Types of participants: patients of all ages with and without cardiovascular comorbidities, undergoing arterial stiffness assessment;
- Follow-up duration: without restrictions;
- Outcome: acute and chronic arterial stiffness changes with BFR training.

Reviews, case reports, studies available only as abstracts (including conference abstracts) and dissertations were excluded from this analysis.

### 2.2. Arterial Stiffness Assessment

We selected studies evaluating arterial stiffness as well as other parameters of vascular function.

Primary indicator: arterial stiffness assessment (pulse wave velocity: PWV; augmentation index: AIx; cardio-ankle vascular index: CAVI; ankle-brachial ratio: ABI).

Secondary indicators: endothelial dysfunction assessment (flow-mediated dilation: FMD).

## 3. Results

We identified a total of 11 literature reports compatible with the beforementioned selection criteria: four studies that assessed the acute influence of BFR training on arterial stiffness (Table 1) and seven studies that examined the medium-to-long-term impact on BFR on vascular function (Table 2).

The acute impact of BFR training on arterial stiffness was studied in small populations of young, healthy individuals. AIx was analyzed before, during and post- 10–55 min of exercise. Rossow et al. [19] reported that AIx decreases during BFR training (more substantially when using a wide cuff) but returns to baseline 15 min after exercise. Figueroa et al. [20] reported a decrease in AIx which persisted 30 min after lower-body resistance training (with and without BFR). Contrary to these results, in two separate studies [21,22], Tai et al. documented increases in AIx after upper- and lower-body resistance training, with or without BFR, which persisted 10 and 25 min post-exercise. At 10 min post-exercise, AIx increased more with upper- versus lower-body training (with or without BFR) and at 25 min post-exercise AIx increased more with upper-body training without BFR versus upper-body training with BFR [21].

We identified seven studies that assessed the long-term influence of BFR on arterial stiffness. Two of them included young, healthy individuals: Although 4 weeks of low-load lower-body BFR (but not high-load resistance training without BFR) improved PWV by 5% in a small group of healthy young men [6], Clark et al. [23] did not document a significant change in PWV or ABI after 4 weeks of low-load lower-body BFR in a smaller mixed-gender group (14 m, 2 f). A single study included middle-aged adults, in which lower-body low-resistance BFR training increased PWV only in the BFR randomized limb (no significant change in the free flow limb) [24]. Two studies focused on healthy, elderly adults and found no change in CAVI, FMD, or ABI after 12 weeks upper- [7] or lower-body [25] low-load BFR training. And finally, two other studies assessed the impact of low-intensity BFR in healthy, older women and showed no impact on CAVI and ABI after 12 weeks of upper- [26] or lower-body [27] BFR training. 

**Table 1:** Acute influence of BFR training on arterial stiffness.

Authors	Population	Study Design	Arterial Stiffness Parameter	Results
Tai et al. [21]	Young individuals performing regular resistance training (≥3 days/week for at least 1 year) N = 23 (14 m, 9 f)	Non-randomized Pulse wave reflections assessed at rest, 10, 25, 40 and 55 min after upper- and lower-body resistance training with and without BFR Upper-body training—latissimus dorsi pulldown and chest press Lower-body training—leg extensions and leg curls Regular training (no BFR) protocol—four sets of eight repetitions at 70% of 1RM with 60 s and 2 min rests between sets and exercises BFR protocol—30-15-15-15 repetitions at 30% of 1RM, with 30 s and 2 min of rest between sets and exercises Cuff pressure—40% AOP, maintained during rest intervals Arterial stiffness assessed before training and post-training (after 10, 25, 40, and 55 min of rest in supine position)	AIx, Aix75 (SphygmoCor XCEL, AtCor Medical, Sydney, Australia)	Upper-body resistance training with or without BFR significantly increase AIx and AIx75 At 10 min post exercise—AIx and AIx75 increased more with upper-body RE +/− BFR versus lower-body RE +/− BFR, At 25 min post-exercise—AIx75 increased more with upper-body RE without BFR versus upper-body RE with BFR. Upper-body RE without BFR also induced higher AIx75 at 25 min post-exercise compared to upper-body RE with BFR. No absolute values provided in the manuscript
Tai et al. (b) [22]	Young men performing regular resistance training (≥3 days/week for at least 1 year) N = 16	AIx and AIx75 assessed in controls and 10 min after low-load BFR and high-load resistance training Low-load BFR training consisted of four sets of 30, 15, 15, and 15 bench press repetitions at 30% of 1RM (30 s rest between sets) The high-load training consisted of four sets of eight Bench press repetitions at 70% of 1RM (1 min rest between sets) For the control measurements, the participants rested in the supine position for 10 min, in order to match the body position of the resistance exercises The tension of the wrap was determined using a visual analog scale of perceived pressure (7 out of 10).	AIx, AIx75 (SphygmoCor XCEL, AtCor Medical, Sydney, Australia)	AIx, AIx75 increased after low-load BFR and after high-load training compared to rest and control Low-load BFR and high-load resistance training resulted in similar increases in AIx and AIx75 No absolute values provided in the manuscript
Rossow et al. [19]	Young healthy individuals N = 27 (13 m, 14 f)	Randomized cross-over study AIx and AIx75 measured at rest, upon inflation, mid-exercise, immediately post-, 5 min and 15 min post-exercise. Participants performed two separate BFR training sessions at 20% of 1RM with two different cuffs (narrow-elastic and wide-non-elastic) The exercise protocol consistent of four sets of knee extension at 20% of 1RM and: 30 repetitions, 30 s rest, 15 repetitions, 60 s rest, 15 repetitions, 30 s rest, 15 repetitions. Cuff pressure was 130% of resting SBP. Cuffs remained inflated throughout the exercise session (including rest periods).	AIx (SphygmoCor, AtCor Medical, Sydney, Australia)	AIx decreased during BFR but returned to baseline 15 min after exercise. Wide cuff use was associated with a more substantial decrease in AIx: Wide cuff—AIx decreased from 9% to −4% mid-exercise and to −9% immediately post-exercise Narrow cuff—AIx decreased from 6%, to 1% mid-exercise and to 0% immediately post-exercise
Figueroa et al. [20]	Young, physically active, healthy subjects N = 23 (12 f, 11 m)	Prospective, randomized cross-over AIx measured at baseline, 2 and 30 min post-exercise Participants performed three separate sessions—control session (no training), low-intensity resistance exercise (40% of 1RM) and low-intensity resistance training with BFR During control, subjects rested on the seated leg extension machine. Training protocol consisted of seated bilateral leg flexion and extension at 40% of 1RM, performed until fatigue, with a 1 min inter-set rest, with and without BFR. Cuff pressure was set at 100 mmHg. Cuffs were deflated during rest periods.	AIx (SphygmoCor, AtCor Medical, Sydney, Australia)	No significant change in AIx during the control session In the low-intensity resistance training group without BFR AIx decreased from 7% (baseline) to −6% (30 min post exercise) In the BFR training group, AIx decreased from 4% (baseline) to −4% (30 min post exercise)

BFR: resistance training with blood flow restriction; 1RM: one-repetition maximum; SBP: systolic blood pressure; N: number; m: male; f: female; HLRT: high-load resistance training; AOP: arterial occlusion pressure; RE: resistance exercise; AIx: augmentation index; AIx75: augmentation index at 75 beats per minute.

**Table 2:** Long-term influence of BFR training on arterial stiffness.

Authors	Population	Study Design	Arterial Stiffness Parameter	Results
Horiuchi et al. [6]	Healthy young men (18–30 years) N = 24 (12—low-load BFR, 12—high load resistance training no BFR)	Prospective randomized control trial haPWV measured before training, at 2 weeks and after 4 weeks of training In the HLRT group, participants performed bilateral knee extensions and leg presses (75% of 1RM)—3 × 10 repetitions with 2 min rest intervals, 4 days/week, for 4 weeks In the BFR group, participants performed low-intensity (30% of 1RM) bilateral knee extensions and leg presses, 4 × 20 repetitions with 30 s rest intervals, 4 days/week, for 4 weeks haPWV assessed before training and after 2 and 4 weeks of training. Occlusive pressure was set at 1.3× SBP. The cuff was inflated during the entire training session	haPWV Vasera-1000 (Fukuda-Denshi Co., Ltd., Tokyo, Japan)	haPWV improved by 5% after BFR (Δ − 0.32 m/s), 95% CI (−0.51–−11.8) haPWV did not significantly vary after HLRT (+1% (Δ + 0.03 m/s), 95% CI (−0.17–0.23)
Yasuda et al. [7]	Healthy elderly adults aged 61–85 years (low load-BFR N = 7, low load resistance training without BFR N = 7)	Prospective trial FMD and CAVI assessed before the start of the study and 3–7 days after the 12 weeks training period Participants performed two training sessions/week. Each session consisted of low-load (30% of 1RM) elastic band bilateral arm curls and triceps press-downs—75 repetitions (30, 15, 15, and 15 repetitions, 30 s rests between each set) for both exercises (90 s rests between different exercises). For the BFR group (seven patients) cuff pressure was gradually inflated from 30 to 120 mm Hg on the first day of training. Cuff pressure was increased by 10–20 mm Hg at each subsequent training session until 270 mm Hg, if tolerated The mean cuff pressure throughout training was 196 +/− 18 mm Hg. Cuff pressure removed after completion of the two exercises	FMD—UNEX EF (Unex Co. Ltd., Nagoya, Japan) CAVI and ABI—VS-1500 system (Fukuda Denshi Co., Ltd., Tokyo, Japan).	No significant change in CAVI (*p* = 0.150), FMD (*p* = 0.116) and ABI (*p* = 0.485) after 12 weeks in either group
Yasuda et al. (b) [26]	Healthy older women (61–85 years old) (low load BFR N = 7, low load training without BFR N = 7)	Prospective randomized trial Arterial stiffness assessed before, after 12 weeks of training, and after 12 weeks of detraining Both groups performed two training sessions/week for 12 weeks. Each session consisted of low-load (30% of 1RM) elastic band bilateral arm curls and triceps press-downs—75 repetitions (30, 15, 15, and 15 repetitions, 30 s rests between each set) for both exercises (90 s rests between different exercises), with or without BFR (according to randomized group allocation) For the BFR group (seven patients) cuff pressure was gradually inflated from 30 to 120 mm Hg on the first day of training. Cuff pressure was increased by 10–20 mm Hg at each subsequent training session until 270 mm Hg, if tolerated The mean cuff pressure throughout training was 202 +/− 8 mm Hg. Cuff pressure removed after completion of the two exercises During the detraining period (12 weeks), participants stopped resistance training, and returned to their normal daily activities as prior to the resistance training period During the 12 weeks detraining period, participants returned to their normal daily activities	FMD—UNEX EF (Unex Co. Ltd., Nagoya, Japan) CAVI and ABI—VS-1500 system (Fukuda Denshi Co., Ltd., Tokyo, Japan).	No significant changes in arterial FMD, CAVI and ABI over the duration of the study.
Yasuda et al. (c) [25]	Healthy elderly subjects, 61–84 years old (BFR training N = 9, control (no training) N = 10)	Prospective randomized Vascular function assessed before and 3–7 days after the final training session. The BFR training group performed two training sessions/week for 12 weeks. Each session consisted of low-load knee extensions (20% of 1RM) and leg press exercises (30% of 1RM)—75 repetitions (30, 15, 15, and 15 repetitions), 30 s rests between each set, for both exercises (90 s rests between different exercises) Cuff pressure was set at 120 mm Hg for the first day of training and then gradually increased by 10–20 mm Hg at each subsequent training session until 270 mm Hg, if tolerated Cuffs remained inflated during both exercises and rest periods	FMD—UNEX EF (Unex Co. Ltd., Nagoya, Japan) CAVI and ABI—VS-1500 system (Fukuda Denshi Co., Ltd., Tokyo, Japan).	No significant change in CAVI and ABI in either group. FMD tended to improve in BFR group (2.8 +/− 2.0%, 4.4 +/− 2.5%, *p* = 0.09).
Yasuda et al. (d) [27]	Healthy, physically active elderly women, 61–86 years old (low-intensity BFR N = 10, middle to high-intensity training N = 10, no training N = 10)	Prospective, randomized Vascular function assessed before and 3–7 days after the final training session Participants randomized to low-intensity BFR or middle to high-intensity resistance training performed squat and elastic bands knee extension exercises, 2 days/week for 12 weeks Training protocol in he low-intensity BFR (35–45% of 1RM) group consisted of 75 repetitions (30, 15, 15, and 15). A 30 s resting period between sets was allocated for both exercises and a 90 s rest interval was allocated between the two exercises In the middle- to high-intensity group (70–90% of 1RM) the training protocol consisted of 37—38 repetitions (13, 13 (from the 1st to the 12th training session) or 12 (from the 13th to the 24th training session), and 12). A 30 s rest period between sets was allocated for both exercises and a 90 s rest interval was allocated between the two exercises For the BFR group cuff pressure was gradually inflated from 50 to 120 mm Hg on the first day of training. Cuff pressure was increased by 10–20 mm Hg at each subsequent training session until 200 mm Hg, if tolerated The mean cuff pressure throughout training was 161 +/− 12 mm Hg. Cuffs remained inflated during both exercises and rest periods.	CAVI and ABI–VS-1500 system (Fukuda Denshi Co., Ltd., Tokyo, Japan). Central AIx (HEM-9000AI, Omron Healthcare Co., Ltd., Kyoto, Japan)	No significant change in central AIx, ABI and CAVI in either group.
Fahs et al. [24]	16 middle-aged adults 40–64 years old (11 m, 5 f) performing lower body low-load resistance training (one limb BFR training, one limb free flow training)	Prospective randomized PWV measured 3 weeks and 1 week before training and 48–96 h after the last training session Participants performed three sessions of training per week for 6 weeks. The training protocol consisted of low-load 30% of 1RM knee extensions, performed in sets of 20 repetitions/minute, to volitional fatigue. For each patient, one limb was randomized to BFR training and one limb to free flow training. For the first 2 weeks, participants completed two sets of exercise per limb per session. During weeks 3–4 participants completed three sets of exercise per limb per session. During weeks 5–6 participants completed four sets of exercise per limb per session. One min rest intervals were allocated between all sets. The order of training (BFR first versus free flow first) alternated with each session During the first week of training, the cuff pressure was set at 150 mmHg or 50% of AOP. During the following weeks cuff pressure was set at 80% AOP (no higher than 240 mmHg). The cuff remained inflated during the entire training session	Femoral PWV (Sphygmocor, Atcor Medical, Sydney, Australia)	BFR limb (PWV increased from 8.9 (0.8) to 9.5 (0.9) m/s), *p* < 0.05 Free flow limb—no significant change in PWV
Clark et al. [23]	Young, healthy adults N = 16 (14 m, 2 f) randomized to high load resistance training (N = 5) and low load BFR training (N = 9)	Prospective, randomized PWV measured before training and 2–3 days after training completion Participants performed three sessions of training per week for 4 weeks Participants randomized to high-load resistance training (N = 5) performed 8–12 bilateral knee extensions at 80% of 1RM to volitional failure, with 90 s rest between each set Participants randomized to low-load BFR training (N = 9) performed 8–12 bilateral knee extensions at 30% of 1RM to volitional failure, with a 90 s rest between each set. The cuff pressure was set at 130% resting brachial SBP. The cuff pressure was maintained throughout the entire exercise session	Femoral-tibial PWV (Biopac MP150 Systems, Goleta, CA, USA) ABI (MD6 System, D.E. Hokanson Inc., Bellevue, WA, USA)	No significant changes in PWV or ABI following training for either group (*p* > 0.05).

BFR: resistance training with blood flow restriction; N: number; m: male; f: female; 1RM: one-repetition maximum; HLRT: high-load resistance training; haPWV: heart-ankle pulse wave velocity; ABI; ankle-brachial pressure index; FMD: flow mediated dilation; CAVI: cardio-ankle vascular index; AIx: augmentation index; PWV: pulse wave velocity; SBP: systolic blood pressure; AOP: arterial occlusion pressure.

## 4. Discussion

Ageing is naturally associated with a certain degree of arterial stiffening, explained by degenerative changes in the arterial wall which accelerate with age (elastin degradation, increase in collagen fibers, and calcification of the aortic media). PWV (inversely related to arterial distensibility) and AIx (a composite parameter that varies with the site and degree of wave reflection) exhibit a non-linear, age-related increase that becomes more prominent after the fifth decade [28]. AIx and PWV are considered more sensitive arterial stiffening markers for young and old adults, respectively [28].

Exercise training improves vascular structure and function [29] and current guidelines recommend both endurance and resistance training for cardiovascular prevention. Long-term high-load resistance training (60–70% of 1RM) promotes muscular fitness, improves lipid profile, and cardiovascular morbi-mortality with a less consistent effect on brachial blood pressure values [30]. However, elderly patients are generally unable to withstand high mechanical stress and are usually prescribed a lower-intensity training protocol [3], which is less effective in correcting sarcopenia, a common finding in heart failure and geriatric populations. Although aerobic training improves arterial stiffness and is an essential instrument in cardiovascular prevention, it does not correct sarcopenia [31]. Muscle loss is addressed by prescribing resistance (strength) training at moderate to high intensities. These are not easily tolerated by elderly patients and transiently reduce central artery compliance even in young, healthy men [32] and are usually avoided in geriatric patients with associated cardiovascular disease. However, low-intensity BFR with moderate vascular restriction (100 mmHg) results in similar muscle adaptations at low intensities (20% of 1RM) [7], explaining the emerging interest in BFR as a critical rehabilitation tool in cardiovascular patients.

For this reason, BFR exercises have emerged as a promising alternative to standard strength training especially for elderly, untrained subjects and those with orthopedic and musculoskeletal impairments. BFR training is performed using a pneumatic cuff inflated in the proximal segment of the exercising limb. The occlusive pressure usually set at 1.3 times of individual SBP [6]. The inflated cuff restricts arterial flow and venous return, inducing local metabolic stress and central hemodinamic changes. Low-intensity BFR (20–30% of 1RM) is similar to standard high-intensity resistance training in increasing muscle mass and strength, independent of age [33]. Research regarding BFR resistance training is sparce, but promising in respect to safety outcomes, with an emerging number of studies focusing on the acute and long-term effects on BFR on arterial stiffness parameters.

### 4.1. Potential Risks with BFR Resistance Training

Sedentarism has been associated with increased all-cause and cardiovascular mortality, and increased risk of oncologic, cardio-metabolic (dyslipidemia, hypertension, diabetes) and neuropsychiatric complications [34]. However, exercise protocols are prescribed with caution in frail patients in order to avoid adverse outcomes. While regular physical activity favors fibrinolysis, high-intensity exercise may induce a prothrombotic state. This risk could be augmented with BFR, as blood lactate correlated with thrombin-antithrombin III complex concentrations and tissue plasminogen activity peak after BFR [2]. Furthermore, BFR training could cause fine microvascular damage (supported by the slight elevations in IL-6 observed after vascular occlusion) which may trigger local thrombosis [35]. However, D-dimer and fibrin/fibrinogen degradation products do not increase in older adults performing BFR [7,36]. Another concern is the potential tissue damage associated with prolonged hypoxia. Indeed, BFR leads to venous congestion and distention, and potential damage to venous valves. However, Takarada et al. [35] showed that light resistance training (20% of 1RM) combined with occlusion (214 mmHg) does not induce considerable tissue damage (assessed via creatine phosphokinase activity and lipid peroxide levels). Muscle damage can occur after unaccustomed exercise involving a large amount of eccentric contractions, but low-intensity BFR has not been associated with increased creatine phosphokinase or myoglobin concentrations [2], even in older adults [7].

Although thrombotic complications are rarely associated with BFR (a rate of 0.06%, which is lower than thrombosis incidence in the general population) [37] candidates should undergo regular coagulation and blood pressure monitoring, with special attention regarding deep venous thrombosis risk [38]. Diabetes, arterial hypertension, chronic kidney disease, rheumatoid arthritis, cancers, thrombophilia, pregnancy, postpartum, and post-surgery status are associated with higher thrombotic risk. In such cases it is useful to use Caprini or IMPROVE scales and exclude high-risk patients from BFR resistance training [38].

As with all other forms of exercise training, individuals with type 1 diabetes performing BFR training should apply routine screening precautions in order to avoid post-exercise hypoglycemia. These include pre- and post-exercise glycemia checks, assessing ketone levels and adjusting carbohydrate intake before and after exercise [38].

Impaired exercise capacity is a both risk factor and a result of chronic kidney disease. Individuals with end-stage chronic kidney disease are more prone to cardiovascular complications, fragility fractures and musculoskeletal pain and should be gradually and cautiously exposed to BFR. Furthermore, electrolyte imbalances, pulmonary congestion, peripheral edema and excess inter-dialytic weight gain are formal contraindications for exercise training in this population [38].

Compared to free flow exercises, BFR training significantly increases plasma adrenaline concentration and should not be prescribed to patients with recent cerebrovascular events. Skeletal muscle contraction activates the exercise pressor reflex (EPR), which enhances sympathetic nervous response with subsequent hemodynamic implications [39]. High-intensity resistance training at 70–100% of 1RM leads to a significant increase in thoracic pressure and a quasi-complete occlusion of skeletal muscular blood flow due to peripheral mechanical compression. As such, high-intensity resistance training leads to an acute increase in HR, systolic, diastolic, and mean arterial pressure, causing significant hemodynamic strain, proportional with the number of repetitions per set [40].

Despite similar individual perceptual responses, HR and BP (especially diastolic blood pressure) increase to a greater extent during BFR training compared to low- and moderate-intensity strength training [41]. In BFR training the exercise pressor reflex is exacerbated by the mechanical vasculature compression, which is higher than the endogenous muscular compression obtained with high-resistance training [39]. BFR training reduces venous return, causing a decrease in systolic volume. However, cardiac output increases due to a marked increase in HR and cardiac workload. The reduced venous return (cardiac preload) observed with BFR can prove useful in patients with associated cardiac disease [37]. The hemodynamic changes observed after BFR are transient, as HR and BP both naturally decrease 30–60 min after training, a similar pattern to that observed with high-load resistance training [15].

Acute cardiovascular changes in low-load BFR training are similar for young and healthy older adults [42]. However, preexistent hypertension is associated with endothelial dysfunction, elevated sympathetic activity, and altered muscle metabo- and mechano-reflexes, explaining the heightened hemodynamic response observed with BFR training [43]. For instance, although BFR without exercise does not significantly impact BP values in healthy young subjects [44], hypertensive elderly women present a mild to moderate pressor response to resting BFR [40]. Abrupt increases in BP increase the risk of cerebrovascular events, raising concerns regarding the safety of this technique in patients with cardiovascular disease. However, a previous analysis of 18 elderly hypertensive females reported similar pressor response after high-load RT and low-resistance BFR [40], recommending equalization of volumes and recovery times in order to minimize BP elevations during exercise [40]. Although two studies have applied BFR training in patients with stable cardiovascular disease with no reported adverse outcomes [4,5], the safety of BFR training in patients with hypertension or associated cardiovascular disease is yet to be determined in larger studies with a longer follow-up.

Nascimento et al. recently published a set of criteria requiring immediate BFR training termination, including (but not limited to) neurological symptoms (confusion, dizziness, impaired balance), nausea or vomiting, significant arrythmia, decrease in SBP or an acute pressor response, chest pain or discomfort suggesting myocardial ischemia, discoloration or significant pain or temperature change in the affected limb [38].

Increased and prolonged activation of the muscle metaboreflex secondary to BFR training may increase BP and illicit abnormal cardiovascular responses (increased retrograde shear stress, intermittent sympathetic overactivation), which raises concerns in prescribing the exercise program to patients with established cardiovascular disease [43]. Indeed, several adverse outcomes, ranging from mild (dizziness, nausea, subcutaneous hemorrhage) [45] to worrisome rhabdomyolysis and central retinal vein occlusion [46] have been reported with BFR training. However, BFR training rarely leads to serious adverse outcomes when performed according to guidelines, in a controlled clinical environment [47].

### 4.2. Peripheral Blood Flow Changes in BFR Resistance Training

From a physiological and cellular perspective, exercise upregulates the activity of arterial endothelial nitric oxide synthase, improving endothelial function and reducing arterial stiffness. In healthy individuals, acute aerobic training reduces central arterial stiffness, wave reflections, and it is postulated that regular aerobic exercise may delay vascular aging. On the other hand, acute bouts of resistance exercise may cause a transient increase in central arterial stiffness [10]. Indeed, even in healthy young adults, traditional strength training (≥60% of 1RM) increases sympathetic activity and endothelin 1 levels, inducing an acute transient increase in arterial stiffness (PWV) [37]. Although chronic high-intensity resistance training increases arterial stiffness by 11.6% [11], low training intensities can reduce brachial-ankle PWV [12]. However, the effect of RT on arterial stiffness is more pronounced in young patients, which inherently have low arterial stiffness parameters, which could yield these results clinically insignificant [11]. Furthermore, the increase in arterial stiffness with high-intensity RT is attenuated with simultaneous aerobic training, supporting current guidelines that recommend a combination of both exercise modalities.

With BFR training, the increased shear stress obtained with cuff deflation and reperfusion mechanically stimulates endothelial nitric oxide synthase increasing NO production [48]. As low-intensity resistance training has a beneficial impact on arterial stiffness [12], it was postulated that chronic low-intensity BFR could have a beneficial effect on peripheral vascular function [48].

As shown by Tai et al. [21], upper- and lower-body resistance training exercised have different consequences on vascular stiffness, which can be explained by the variation in transit time of the returning pulse waveform. Upper-body resistance training, with or without BFR is associated with an acute increase in AIx and AIx75, which persists up to 25 min post-exercise [21,22]. On the other hand, with lower-body training (with or without BFR), a significant increase in AIx75 can be observed 10 min after exercise, but not at 25, 40, and 55 min after exercise. As such, current evidence suggests that lower-body resistance training with or without BFR has a lesser impact on pulse wave reflections [21]. Indeed, two other studies documented decreases in AIx after low-resistance lower-body BFR training [19,20] reported similar decreases in AIx 30 min after low-resistance lower-body RE with or without BFR. As arterial stiffness parameters return to baseline shortly after training, the effect could be explained by post-exercise vasodilation [19].

The study of the short- and medium-term impact of BFR resistance training on vascular stiffness has yielded divergent results. Horiuchi et al. [6] showed that 4 weeks of BFR reduces arterial stiffness in healthy young men, as opposed to high-intensity resistance training, which produced the opposite effect. Clark et al. [23] reported no significant change in PWV following 4 weeks of either low-intensity (30% of 1RM) BFR or high-intensity (80% of 1RM) lower-body training in young, healthy adults. On the other hand, Fahs et al. [24] reported a small but statistically significant increase in PWV after 6 weeks of low-load BFR in healthy, middle-aged adults. And lastly, several studies performed in older adults showed that 12 weeks of low-load resistance training (with and without BFR) did not significantly alter vascular function (assessed via CAVI, FMD, and ABI) [7,25,26,27].

Fahs et al. [24] showed that 6 weeks of progressive low-load resistance exercises increased arterial stiffness in middle-aged adults with associated cardiovascular comorbidities. The effect could be explained by increased oxidative stress and a subsequent reduction in nitric oxide bioavailability. The increase in peripheral arterial stiffness was more prominent with BFR compared to free flow training. The same study observed an inverse relationship between pre-training PWV and the change in PWV, which could suggest that the increase in arterial stiffness could be an adaptive response to external compression (cuff pressure and muscle contractions). Although the average increase in arterial stiffness was mild (0.6 m/s, 6.7%), this could have significant long-term implications, as each 1 m/s increase in PWV leads to a 13–15% increase in mortality [49].

### 4.3. The Importance of BFR Protocol

The lack of consistency regarding study methodologies and protocols, especially regarding BFR pressures, poses a significant limitation in comparing the results of previous stud=ies.

As shown by Rossow et al. [19], cuff type impacts training outcome, since cardiovascular responses, ratings of perceived exertion and pain are higher with the use of wider, non-elastic cuffs. The authors reported a higher decrease in AIx during BFR with wide cuff use, although arterial stiffness parameters returned to baseline 15 min after exercise [19].

Previous studies have used different protocols regarding applied cuff pressure. Limb occlusion pressure (LOP) and a more personalized approach, is the current guideline-recommended approach in BFR training [47]. LOP provides a more objective way to implement BFR training and understand its long-term effects on vascular function. LOP is also considered to have a lesser risk of acute exercise-related adverse events, especially in high-risk patients.

Another important protocol variation is the implementation of continuous versus intermittent pressure during exercise. When using LOP, both continuous and intermittent BFR provide similar grades of muscle hypertrophy [50,51]. Maintaining cuff pressure during rest intervals increases post-exercise release of noradrenaline and is associated with a heightened brachial blood pressure increase [37]. Intermittent BFR requires cuff deflation during rest periods and is the preferred method for patients with associated risk factors, as it reduces the acute hemodynamic stress to BFR [52], including arterial stiffness measures [20]. With continuous pressure, Rossow et al. [19] noted a decrease in AIx after cuff inflation and that persisted during exercise, but returned to baseline values 5 min post-exercise. However, in another study which used intermittent BFR [20], AIx dropped below baseline 30 min post-exercise, emphasizing the importance of protocols.

## 5. Conclusions

Despite the increasing number of reports that study the effects of BFR training on vascular function, evidence regarding the long-term effects of BFR remains scarce and no firm recommendation can be made at this point. Furthermore, interpretation of the current literature data is limited by the wide variation in sample sizes, population characteristics, but also BFR protocols (cuff pressure, number of repetitions, training duration, etc.).

Overall, it seems that BFR has a mild or neutral long-term impact on arterial stiffness. However, current research shows that BFR can cause an abrupt, albeit transient, increase in PWV and central blood pressure, even in healthy young people. This effect seems to be more prominent in elderly and hypertensive individuals with an exacerbated muscle metaboreflex pressor response. BFR and, preferably, lower-body BFR, should be prescribed with caution in older populations with preexisting cardiovascular comorbidities.

Further research should focus on developing safe BFR protocols regarding potential moderator variables (age, sex, cuff pressure, training frequency, and intensity) and on the long-term follow-up of vascular stiffness variations with BFR training.
